# The effects of purslane consumption on glycemic control and oxidative stress: A systematic review and dose–response meta‐analysis

**DOI:** 10.1002/fsn3.3311

**Published:** 2023-03-15

**Authors:** Naser Jafari, Nargeskhatoon Shoaibinobarian, Azadeh Dehghani, Amirhosein Rad, Seyedeh Nooshan Mirmohammadali, Mohammad Javad Alaeian, Yasaman Hamedi, Mohamad Zamani, Mohammad Ali Goudarzi, Omid Asbaghi

**Affiliations:** ^1^ University of Applied Science and Technology ‐ Allameh Tabarsi Center Tehran Iran; ^2^ Department of Nutrition, School of Medical Sciences and Technologies Islamic Azad University, Science and Research Branch Tehran Iran; ^3^ Nutrition Research Center, Department of Community Nutrition, Faculty of Nutrition and Food Science Tabriz University of Medical Sciences Tabriz Iran; ^4^ Nutrition Department, Faculty of Health and Nutrition Lorestan University of Medical Sciences Khoramabad Iran; ^5^ Department of Food, Nutrition, Dietetics and Health Kansas State University Manhattan Kansas USA; ^6^ MD graduated from Shahid Beheshti University of Medical sciences Tehran Iran; ^7^ Department of Physical Education Central Tehran Branch, Islamic Azad University Tehran Iran; ^8^ Department of Clinical Nutrition, School of Nutritional Sciences and Dietetics Tehran University of Medical Sciences Tehran Iran; ^9^ Shahrekord Branch Islamic Azad University Shahrekord Iran; ^10^ Cancer Research Center Shahid Beheshti University of Medical sciences Tehran Iran

**Keywords:** glycemic, meta‐analysis, oxidative stress, purslane, systematic review

## Abstract

Purslane (*Portulaca oleracea L*.) is a herbal remedy with wide range of pharmaceutic properties. Although the beneficial effect of purslane on the treatment of Type 2 Diabetes Mellitus (T2DM) has been shown, there is an inconsistency among the results of previous studies. Therefore, this study is aimed at conducting a systematic review and meta‐analysis on the effect of purslane on glycemic profile and oxidative stress markers. A systematic search was performed in the Scopus, Web of science, PubMed and the Cochrane Library to find articles related to the effect of the purslane on Malondialdehyde (MDA) and Total Antioxidant Capacity (TAC), Fasting Blood Sugar (FBS), Hemoglobin A1c (HbA1c), insulin resistance, Homeostatic Model Assessment for Insulin Resistance (HOMA‐IR) up to September 2022. Among the 611 initial studies that were identified from searching electronic databases, 16 Randomized Clinical Trials (RCTs) involving 1122 participants (557 cases and 565 controls) were included for data analysis. The results of random‐effects modeling demonstrated that purslane consumption significantly reduced FBS (*p* < .001), MDA (*p* < .001) and increased TAC (*p* < .001). However, purslane consumption did not affect HbA1c (*p* < .109), fasting insulin (*p* = .298) and HOMA‐IR (*p* = .382). Meta‐analyses were performed using both the random‐ and fixed‐effects model where appropriate, and *I* ^2^ index was used to evaluate the heterogeneity. This meta‐analysis study suggests that purslane has beneficial effects on oxidative stress markers and glycemic parameter. Therefore, it may be a promising adjuvant therapy in T2DM because of its benefits and negligible adverse effects.

## Graphical abstract

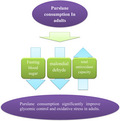

## 1 INTRODUCTION

Diabetes is a common metabolic disease that is characterized by prolonged hyperglycemia and abnormality in protein and lipid metabolism (DeFronzo et al., 2015). The prevalence of diabetes is rising globally and by 2030, there will have been over 360 million patients (Zheng et al., 2018). Common diabetes health complications are neuropathy, nephropathy, retinopathy, and cardiovascular disease (CVD) (Stolar, 2010). Additionally, one of the major problems with managing diabetes is the high expense of therapy (Domeikienė et al., 2014). Patients with diabetes are more likely to experience higher levels of oxidative stress due to increased generation of reactive oxygen species (ROS) and weakened antioxidant defense mechanisms (Oguntibeju, 2019). Hyperglycemia can lead to oxidative stress (Luc et al., 2019) which decreases insulin secretion (Maddux et al., 2001). Decreased oxidative stress may therefore aid in diabetes‐related challenges (Piconi et al., 2003). Synthetic diabetic medications should not be taken during pregnancy due to their potential adverse effects (El‐Sayed, 2011). Therefore, taking herbal antidiabetic agents could be beneficial (Asbaghi et al., 2019, 2021, 2022). *Portulaca oleracea* known as purslane is one of the herbs that can reduce blood glucose (Gong et al., 2009; Papoli, Pishdad, Nadjarzadeh, & Hosseinzadeh, 2019a; Wainstein et al., 2016), oxidative stress (Elahe Zakizadeh et al., 2013), and has beneficial impacts on decreasing blood total cholesterol and low‐density lipoprotein (LDL) cholesterol (Papoli et al., 2019; Sabzghabaee et al., 2014; Zakizadeh et al., 2015). Purslane is a rich source of omega 3, carotene, flavonoids, glutathione, phenolic components, amino acids, tocopherol, polysaccharides, vitamin B complex and some other minerals and active biologic compounds such as dopamine and noradrenaline (Gheflati et al., 2019; Ghorbanian et al., 2019; PARVIN et al., 2013) In addition to the previously mentioned benefits, purslane has positive effects on scavenging free radicals, aiding total antioxidant capacity (TAC), treating various types of cancer and CVD (L. Liu et al., 2000; Yang et al., 2009).

Consuming 10 g of purslane seeds daily for 8 weeks had similar effects on fasting blood sugar (FBS) and serum insulin levels to taking 1500 mg of metformin daily (Esmaillzadeh et al., 2015). The leaves and seeds of purslane have additional health benefits including analgesic, neuroprotective, wound healing, and bronchodilation (Darvish Damavandi et al., 2021; Gheflati et al., 2019). Since to the best of our knowledge, there was no prior systematic review and dose–response meta‐analysis investigating the effects of purslane on glycemic control and oxidative stress, this study was conducted to summarize the evidence from the literature regarding the effect of purslane intake on glycemic control and oxidative stress.

## 2 MATERIALS AND METHODS

### 2.1 Literature search

The Preferred Reporting Items for Systematic reviews and Meta‐Analyses (PRISMA) guidelines for the development of protocols and reporting of systematic reviews and meta‐analyses were conformed (Page et al., 2021). The following databases were used to find relevant Randomized Clinical Trials (RCTs) and recent systematic reviews of purslane in adults up to September 2022: PubMed, the Cochrane library, and Scopus and ISI web of science. The keywords (“Purslane” OR “Portulaca” OR “Portulaca oleracea”) AND (intervention OR “Intervention Study” OR “Intervention Studies” OR “controlled trial” OR randomized OR randomized OR random OR randomly OR placebo OR “clinical trial” OR trial OR “randomized controlled trial” OR “randomized clinical trial” OR RCT OR blinded OR “double blind” OR “double blinded” OR trial OR “clinical trial” OR trials OR “Pragmatic Clinical Trial” OR “Cross‐Over Studies” OR “Cross‐Over” OR “Cross‐Over Study” OR parallel OR “parallel study” OR “parallel trial”) were used. There were no restrictions on date and language in the searches of different databases. References from review papers were manually searched to find missed studies from initial database search.

### 2.2 Study selection and eligibility criteria

Two authors (AD and OA) revised the titles, abstracts, references, and full texts of related articles to select relevant studies. The inclusion criteria were: (1) adult participants >18 years taking purslane supplementations for ≥2 weeks; (2) having a control group where the only difference between the trial and control groups was the supplementation of purslane; (3) reports of purslane supplementation effects on FBS, Hemoglobin A1c (HbA1c), fasting insulin, Homeostatic Model assessment for Insulin Resistance (HOMA‐IR), Total Antioxidant Capacity (TAC), and Malondialdehyde (MDA); (4) having a RCT design; and (5) purslane not being manipulated as part of a multicomponent supplement in either of the trial or control group. Trials on children, pregnant women, animals, in addition to review papers and case studies were excluded.

### 2.3 Risk of bias

The quality of eligible studies was evaluated using the Cochrane risk of bias tool for RCTs. Two independent investigators (OA and NJ) used the following checklist for each included manuscript to categorize them into three groups of low, moderate, and high risk of bias; (1) sufficient sequence generation, (2) allocation concealment, (3) blinding of all procedures and staff, (4) unbiased appraisal of consequences, (5) incomplete data, (6) optional reporting of results (reporting bias), and (7) other possible sources of bias.

### 2.4 Data extraction

The following data were extracted from included studies by the two authors (NJ and OA) separately: study characteristics (the first author, year of publication, country, study design, study duration, sample size in each group, intervention type and dose), participants' characteristics (sex, mean age, body mass index (BMI)), as well as the mean and standard deviation (SD) of FBS, HbA1c, fasting insulin, HOMA‐IR, TAC and MDA concentrations in the pre‐intervention and post‐intervention phase.

### 2.5 Statistical analysis

We fulfilled this meta‐analysis using STATA statistical software (version 14; STATA Corp LP). Treatment effects were appointed as the weighted mean differences (WMD) and 95% confidence intervals (CIs) were distinguished using the random‐effect models, following the DerSimonian and Laird methods. We calculated changes in FBS, HbA1c, fasting insulin, HOMA‐IR, TAC, and MDA concentrations between the trial and control groups from baseline to the end of the intervention procedure. Pre‐specified subgroup analyses were performed according to baseline FBS, HbA1c, fasting insulin, HOMA‐IR, TAC and MDA, purslane dosage (≤10 g/day vs. >10 g/day), duration of the intervention (≥8 vs. < 8 weeks), and health status of the participants (diabetic vs. non‐diabetic).

Sensitivity analyses were carried out to evaluate the stability of the outcomes by eliminating one study at a time to notice the impact of individual papers on the pooled effect size. Funnel plots and Egger's regression test were applied to discern publication bias. A *p*‐value of <.05 was considered statistically significant unless otherwise specified. The potential non‐linear effects of purslane dose (g/day) and intervention duration (weeks) were studied applying the fractional polynomial modeling as well as administrating meta‐regression to find the confounders and linear relations between the effect size and sample size, duration and intervention dosage.

### 2.6 Certainty assessment

The total certainty of evidence across the studies was scored according to the Grading of Recommendations Assessment, Development, and Evaluation (GRADE) guidelines Working Group. According to the corresponding assessment criteria, the quality of the document was classified into high, moderate, low, and very low categories.

## 3 RESULTS

### 3.1 Study selection

**FIGURE 1:**
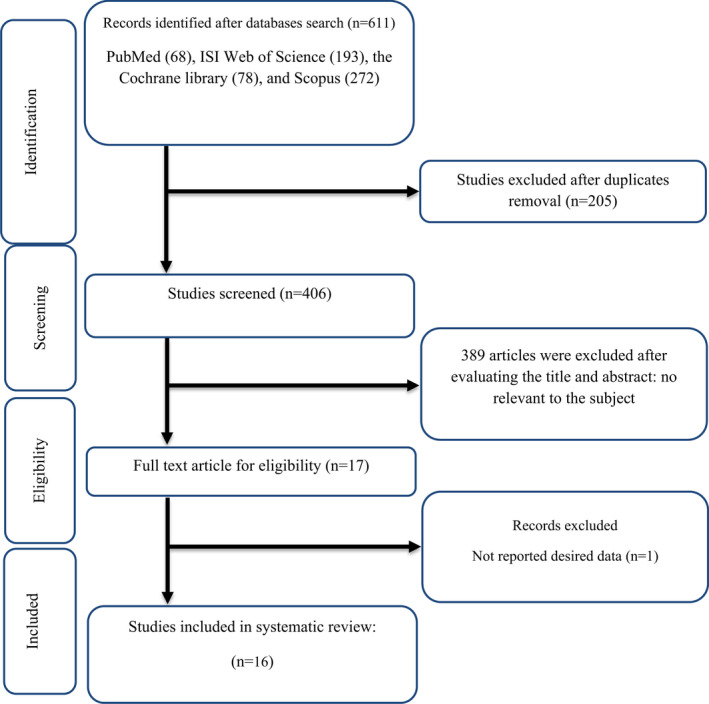
Flow chart of study selection for inclusion trials in the systematic review.

We found 791 publications in Scopus (300), PubMed (236), Cochrane library (89), and ISI Web of Science (166) in the initial search. Of these, 204 articles were duplicated. Thus, a total of 587 articles were assessed for the title and abstract screening. After screening of title and abstract, 570 unrelated studies were discarded due to primary evaluation of inclusion criteria. As a result, 17 studies were retrieved for full‐text review, one of which was excluded due to lack of sufficient data. Consequently, 16 RCTs (Adelnia Najafabadi et al., 2015; Darvish Damavandi et al., 2021; Dehghan et al., 2016; Delvarianzadeh et al., 2021; El‐Sayed, 2011; Esmaillzadeh et al., 2015; Fakoori Jouibari et al., 2014; Farzanegi, 2014; Gheflati et al., 2019; Ghorbanian et al., 2019; Marjan Bedakhanian et al., 2017; Moradi et al., 2012; Papoli et al., 2019; Rafiee Vardanjani et al., 2013; Wainstein et al., 2016; Zakizadeh et al., 2015) were eligible for this systematic review and meta‐analysis. Figure 1 shows the flow chart of the literature search.

### 3.2 Study characteristics

Seventeen RCTs assessing the effects of purslane supplementation on glycemic control and oxidative stress were identified. Included studies were conducted in various countries such as Iran (*n* = 14) (Adelnia Najafabadi et al., 2015; Darvish Damavandi et al., 2021; Dehghan et al., 2016; Delvarianzadeh et al., 2021; Esmaillzadeh et al., 2015; Fakoori Jouibari et al., 2014; Farzanegi, 2014; Gheflati et al., 2019; Ghorbanian et al., 2019; Marjan Bedakhanian et al., 2017; Moradi et al., 2012; Papoli et al., 2019; Rafiee Vardanjani et al., 2013; Zakizadeh et al., 2015), Yemen (*n* = 1) (El‐Sayed, 2011), and Israel (*n* = 1) (Wainstein et al., 2016). All Studies have a parallel design and two of them have a crossover design. Publication dates ranged from 2011 to 2021. The follow‐up periods were from 4 weeks to 16 weeks. The sample sizes for the included studies ranged from 14 to 103. Five studies enrolled only females (Dehghan et al., 2016; Fakoori Jouibari et al., 2014; Farzanegi, 2014; Ghorbanian et al., 2019; Papoli et al., 2019) and the rest of included studies involved both genders (Adelnia Najafabadi et al., 2015; Darvish Damavandi et al., 2021; Delvarianzadeh et al., 2021; El‐Sayed, 2011; Ahmad Esmaillzadeh et al., 2015; Gheflati et al., 2019; Marjan Bedakhanian et al., 2017; Moradi et al., 2012; Rafiee Vardanjani et al., 2013; Wainstein et al., 2016; Zakizadeh et al., 2015). Purslane, purslane and exercise, and purslane with aerobic training were the three types of intervention administered. Eleven studies were conducted on patients with diabetes (Dehghan et al., 2016; Delvarianzadeh et al., 2021; El‐Sayed, 2011; Esmaillzadeh et al., 2015; Fakoori Jouibari et al., 2014; Farzanegi, 2014; Wainstein et al., 2016; Zakizadeh et al., 2015), three studies on patients with non‐alcoholic fatty liver disease (NAFLD) (Darvish Damavandi et al., 2021; Gheflati et al., 2019; Rafiee Vardanjani et al., 2013), two studies on metabolic syndrome (Marjan Bedakhanian et al., 2017; Papoli et al., 2019), one study on patients suffering from schizophrenia (Rafiee Vardanjani et al., 2013), one study on hypercholesterolemic participants (Moradi et al., 2012) and one study on non‐active girls (Ghorbanian et al., 2019). The summary of the characteristics of the included studies is shown in Table 1. Results from the quality assessment are indicated in Table 2.

**TABLE 1:** Characteristic of included studies in meta‐analysis.

Study	Country	Study design	Sex	Trial duration (week)	Participants	Means age		Means BMI		Intervention			Sample size	
						IG	CG	IG	CG	Treatment group	Intervention dose (gr/day)	Control	IG	CG
El‐Sayed (2011)	Yemen	parallel, R, PC, DB	M/F (F: 10, M: 20)	8	Type 2 Diabetes	40 ± 17.52	40 ± 17.52	31.03 ± 3.8	32.27 ± 5.2	purslane	10	metformin	15	15
Moradi et al. (2012)	Iran	Parallel, R	M/F: 93	8	Hypercholesterolemia patients	44 ± 9.6	49 ± 11.6	27 ± 3.9	26 ± 4.9	purslane	50	lovastatin	41	52
Rafiee Vardanjani et al. (2013)	Iran	parallel, R, PC, DB	M/F: 60	8	Schizophrenic Patients	43.76 ± 10.96	45.26 ± 10.03	NR	NR	purslane	1	control diet	30	30
Farzanegi (2014)	Iran	parallel, R, PC	F: 14	8	Type 2 Diabetes	51.17 ± 4.88	50.83 ± 6.79	29.88 ± 4.34	30.71 ± 4.34	purslane & Exercise	7.5	control diet & Exercise	7	7
Farzanegi (2014)	Iran	parallel, R, PC	F: 14	8	Type 2 Diabetes	52.33 ± 4.08	50.17 ± 5.34	29.01 ± 4.34	29.37 ± 4.55	purslane	7.5	control diet	7	7
Fakoori Jouibari et al. (2014)	Iran	parallel, R, PC, DB	F: 14	8	Type 2 Diabetes	50	50	NR	NR	purslane	7.5	control diet	7	7
Fakoori Jouibari et al. (2014)	Iran	parallel, R, PC, DB	F: 14	8	Type 2 Diabetes	50	50	NR	NR	purslane & Exercise	7.5	control diet & Exercise	7	7
Zakizadeh et al. (2015)	Iran	Crossover, R, PC	M/F: 40	5	Type 2 Diabetes	35–65	35–65	NR	NR	purslane	10	control diet	40	40
Esmaillzadeh et al. (2015)	Iran	Crossover, R, PC	M/F: 48	5	Type 2 Diabetes	51.4 ± 6.09	51.4 ± 6.09	28.99 ± 3.9	28.8 ± 3.9	purslane	10	control diet	48	48
Adelnia Najafabadi et al. (2015)	Iran	parallel, R, PC, B	M/F (F: 12, M: 42)	8	Non‐alcoholic fatty liver disease	54.7 ± 9.52	39.891 ± 8.84	32.77 ± 3.63	31.08 ± 3.24	purslane	10	control diet	27	27
Dehghan et al. (2016)	Iran	parallel, R, PC, DB	F: 98	16	Type 2 Diabetes	52.33 ± 4.08	50.17 ± 5.34	29 ± 5	29.9 ± 7.3	purslane	7.5	Placebo	49	49
Dehghan et al. (2016)	Iran	parallel, R, PC, DB	F: 98	16	Type 2 Diabetes	61.17 ± 4.88	58.83 ± 6.79	29.8 ± 6.4	29.5 ± 7.2	purslane & Aerobic Training	7.5	placebo & Aerobic Training	49	49
Wainstein et al. (2016)	Israel	parallel, R, PC, DB	M/F (F: 22, M: 41)	12	Type 2 Diabetes	52.4 ± 7.9	58.3 ± 10.8	29.9 ± 3.8	29.1 ± 3.6	purslane	0.18	placebo	31	32
Marjan Bedakhanian et al. (2017)	Iran	parallel, R, PC	M: 78	8	Metabolic Syndrome	46.5 ± 7.6	47.8 ± 6.5	28.38 ± 1.79	28.57 ± 2.15	purslane	0.06	control diet	39	39
Gheflati et al. (2019)	Iran	parallel, R, PC	M/F (F: 48, M: 12)	8	Non‐alcoholic fatty liver disease	40.07 ± 9.52	39.81 ± 8.84	32.77 ± 3.63	31.09 ± 3.24	purslane	10	control diet	27	27
Ghorbanian et al. (2019)	Iran	parallel, R, PC	F: 20	8	Non‐Active Girls	20–30	20–30	27 ± 2.6	28.21 ± 9.8	purslane	1.2	control diet	10	10
Papoli et al. (2019)	Iran	parallel, R, PC	F: 64	12	Metabolic syndrome	42.16 ± 5.48	43.16 ± 8.33	28.23 ± 4.43	26.3 ± 3.72	purslane	10	control diet	32	32
Darvish Damavandi et al. (2021)	Iran	parallel, R, PC, DB	M/F (F: 31, M: 43)	12	Non‐alcoholic fatty liver disease	46.18 ± 9.71	46.05 ± 10.09	31.56 ± 3.78	31.83 ± 3.97	purslane	0.3	Placebo	37	37
Delvarianzadeh et al. (2021)	Iran	parallel, R, PC, DB	M/F (F: 51, M: 53)	4	Type 2 Diabetes	53.5 ± 6.75	53.6 ± 6.34	NR	NR	purslane	10	control diet	54	50

Abbreviations: CG, control group; F, Female; IG, intervention group; M, Male; NR, not reported; NR, not reported.

**TABLE 2:** Quality assessment.

Study	Random sequence generation	Allocation concealment	Selective reporting	Other sources of bias	Blinding (participants and personnel)	Blinding (outcome assessment)	Incomplete outcome data	General quality
El‐Sayed (2011)	L	L	H	L	L	U	L	Moderate
Moradi et al. (2012)	L	H	H	H	H	H	L	Low
Rafiee Vardanjani et al. (2013)	L	H	H	H	L	U	L	Low
Farzanegi (2014)	L	H	H	H	H	H	L	Low
Fakoori Jouibari et al. (2014)	L	H	H	H	L	U	L	Low
Zakizadeh et al. (2015)	L	H	H	L	H	H	L	Low
Esmaillzadeh et al. (2015)	L	L	H	L	H	H	L	Low
Adelnia Najafabadi et al. (2015)	L	L	H	L	L	H	L	Low
Dehghan et al. (2016)	L	H	H	H	L	U	L	Low
Wainstein et al. (2016)	L	L	L	L	L	U	L	
Marjan Bedakhanian et al. (2017)	L	L	H	H	H	H	L	Low
Gheflati et al. (2019)	L	L	H	L	H	H	L	Low
Ghorbanian et al. (2019)	L	H	H	H	H	H	L	Low
Papoli et al., 2019)	L	L	H	L	H	H	L	Low
Darvish Damavandi et al., 2021)	L	L	H	L	L	U	L	Moderate
Delvarianzadeh et al., 2021)	L	H	H	H	L	U	L	Low

Abbreviations: H, high‐risk of bias; L, low‐risk of bias; U, unclear‐risk of bias.

### 3.3 Meta‐analysis

#### 3.3.1 Effect of purslane supplementation on FBS

**FIGURE 2:**
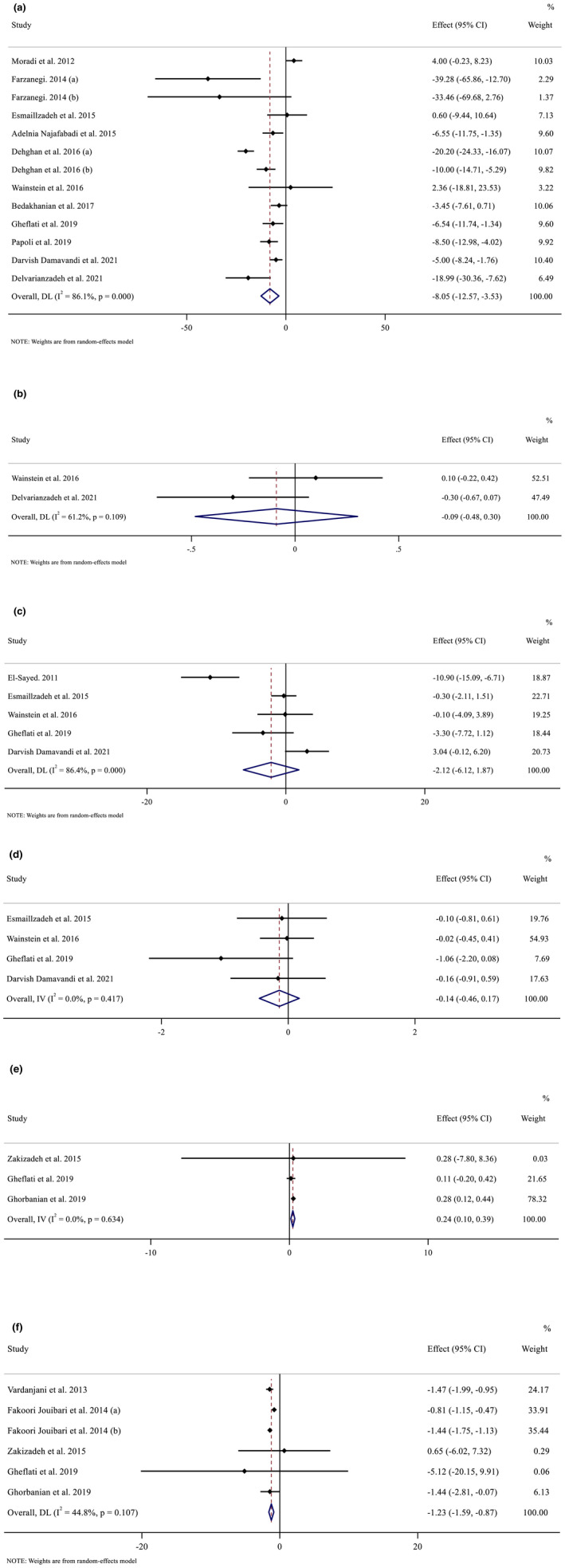
Forest plot detailing weighted mean difference and 95% confidence intervals (CIs) for the effects of purslane consumption on A) FBS (mg/dL); B) HbA1c (%); C) Fasting insulin (μIU/ml); D) HOMA‐IR; E) TAC (mmol/l) and MDA (μmol/ml).

Overall, 13 effect sizes evaluated the effect of purslane supplementation on FBS. Pooled effect size from the random‐effect model showed a significant decreasing effect of purslane supplementation on FBS (WMD: −8.05 mmol/L; 95% CI: −12.57 to −3.53, *p* < .001). There was significant heterogeneity between studies (*I* ^2^ = 86.1%, *p* < .001) (Figure 2a). However, after subgroup analysis, we observed significant effect of purslane supplementation on FBS in studies of less (WMD: −6.32 mmol/L; 95% CI: −11.92 to −0.73, *p* = .027) and more than 8 weeks (WMD: −9.96 mmol/L; 95% CI: −16.49 to −3.44, *p* = .003). Similar significant effects were also detected in female (WMD: −15.43 mmol/L; 95% CI: −22.96 to −7.90, *p* < .001) and both sexes (WMD: −4.04 mmol/L; 95% CI: −7.83 to −0.25, *p* = .036). In addition, sub‐group analysis based on dosage showed that purslane supplementation for <10 g/day had significant effect on FBS (WMD: −11.11 mmol/L; 95% CI: −18.20 to −4.02, *p* = .002). In addition, subgroup analysis demonstrated that purslane affected both diabetic (WMD: −13.74 mmol/L; 95% CI: −21.73 to −5.74, *p* = .001) and non‐diabetic patients (WMD: −4.24 mmol/L; 95% CI: −7.77, −0.71, *p* = .018) significantly. Sub‐group analysis based on baseline BMI showed that purslane supplementation had significant effect on overweight and obese subjects, respectively (WMD: −8.31 mmol/L; 95% CI: −15.34, to −1.29, *p* = .020) (WMD: −5.67 mmol/L; 95% CI: −8.10 to −3.24, *p* < .001). However, after subgroup analysis, we observed significant effect of purslane supplementation on FBS in studies of more than 100 mg/dL baseline serum FBS (WMD: −11.14 mmol/L; 95% CI: −17.28 to −5.01, *p* < 0.001) (Table 3).

**TABLE 3:** Subgroup analyses of purslane supplementation on glycemic control and oxidative stress.

	Effect size	WMD (95%CI)	*p*‐value	Heterogeneity	
				*p* heterogeneity	*I* ^2^
Subgroup analyses of purslane on FBS level.					
Overall effect	13	−8.05 (−12.57, −3.53)	**<.001**	<.001	86.1%
Sex					
Both sexes	8	−4.04 (−7.83, −0.25)	**.036**	.001	70.4%
Female	5	−15.43 (−22.96, −7.90)	**<.001**	<.001	81.6%
Trial duration (week)					
≤8	8	−6.32 (−11.92, −0.73)	**.027**	<.001	78.2%
>8	5	−9.96 (−16.49, −3.44)	**.003**	<.001	88.4%
Intervention dose (g/day)					
≥10	6	−5.37 (−5.37, −10.96)	.059	<.001	81.4%
<10	7	−11.11 (−18.20, −4.02)	**.002**	<.001	87.8%
Baseline BMI (kg/m^2^)					
Overweight (25–29.9)	9	−8.31 (−15.34, −1.29)	**.020**	<.001	90.1%
Obese (≥30)	3	−5.67 (−8.10, −3.24)	**<.001**	.827	0.0%
Health status					
Diabetes	7	−13.74 (−21.73, −5.74)	.001	<.001	77.7%
Non‐diabetes	6	−4.24 (−7.77, −0.71)	.018	.001	74.9%
Baseline serum FBS (mg/dL)					
<100	4	−3.41 (−8.39, 1.55)	.178	.001	80.7%
>100	9	−11.14 (−17.28, −5.01)	**<.001**	<.001	83.1%
Subgroup analyses of purslane on HbA1c.					
Overall effect	2	−0.09 (−0.48, 0.30)	.652	.109	61.2%
Subgroup analyses of purslane on fasting insulin.					
Overall effect	5	−2.12 (−6.11, 1.87)	.298	<.001	86.4%
Subgroup analyses of purslane on HOMA‐IR level.					
Overall effect	4	−0.14 (−0.45, 0.17)	.382	.417	0.0%
Subgroup analyses of purslane on TAC.					
Overall effect	3	0.24 (0.10, 0.38)	**.001**	.634	0.0%
Subgroup analyses of purslane on MDA.					
Overall effect	6	−1.23 (−1.59, −0.86)	**<.001**	.107	44.8%

Abbreviations: BMI, body mass index; CI, confidence interval; FBS, fasting blood glucose; HbA1c, hemoglobin A1c; HOMA‐IR, Homeostatic Model Assessment for Insulin Resistance; MDA, malondialdehyde; TAC, total anti‐oxidant capacity; WMD, weighted mean differences.

Bold values indicate significant effect (*p*‐value < .05).

#### 3.3.2 Effect of purslane on HbA1c

Two clinical trials evaluated the effect of purslane on HbA1c. Pooled effect size from random‐effect model showed nonsignificant decreasing effect of purslane supplementation on HbA1c (WMD: −0.09 mmol/L; 95% CI, −0.48 to 0.30, *p* = .652). There was no significant heterogeneity between studies (*I* ^2^ = 61.2%, *p* < .109) (Figure 2b).

#### 3.3.3 Effect of purslane on fasting insulin

Upon combining five effects from 17 studies, nonsignificant differences in fasting insulin were seen in the intervention compared to the control group (WMD: −2.12 mg/dL, 95% CI −6.11 to 1.87, *p* = .298) following purslane supplementation. The studies were significantly heterogeneous (*I* ^2^ = 86.4%, *p* < .001; Figure 2C).

#### 3.3.4 Effect of purslane on HOMA‐IR

Four effect sizes from 17 studies were included in this meta‐analysis. When compared with the control group counterparts, quantitative meta‐analysis revealed that purslane supplementation had no significant influence on HOMA‐IR levels in the intervention group (WMD: −0.14 mg/dL, 95% CI −0.45 to 0.17, *p* = .382; Figure 2d).

#### 3.3.5 Effect of purslane on TAC

Three effect sizes presenting data on purslane supplementation on TAC were analyzed. Quantitative meta‐analysis revealed a significant weighted mean effect of purslane supplementation on TAC in the intervention group compared with the control group (WMD: 0.24 mg/dL, 95% CI 0.10 to 0.38, *p* < .001) (Figure 2e).

#### 3.3.6 Effect of purslane on MDA

After combining six effect sizes from studies, purslane supplementation significantly reduced MDA when compared with various intervention strategies used on participants to those in control groups (WMD: −1.23 mg/L, 95% CI −1.59 to −0.86, *p* < .001; Figure 2f).

#### 3.3.7 Dose–response analyses

Non‐linear dose–response analyses did not demonstrate a significant relationship between doses and duration and changes in FBS levels (Figures 4 and 5). In addition, Meta‐regression analysis did not indicate a linear relationship between doses (Figure 6). However, results showed a linear relationship between duration and FBS (*p* = .012; Figure 7).

#### 3.3.8 Publication bias and sensitivity analyses

**FIGURE 3:**
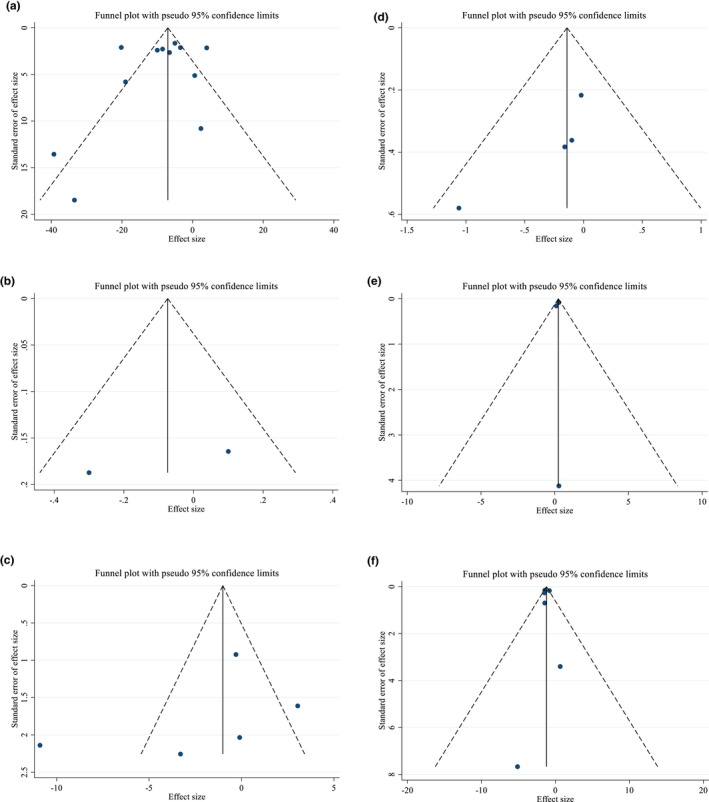
Funnel plots for the effect of purslane consumption on A) FBS (mg/dL); B) HbA1c (%); C) Fasting insulin (μIU/ml); D) HOMA‐IR; E) TAC (mmol/l) and MDA (μmol/ml).

Based on visual inspection of funnel plots and Egger's regression test, no evidence of publication bias for FBS (*p* = .491), fasting insulin (*p* = .461), HOMA‐IR (*p* = .147), TAC (*p* = .747), and MDA (*p* = .856) was found (Figure 3a–f). Therefore, findings from the sensitivity analyses showed no significant effect of any individual study on the entire effect sizes of FBS, fasting insulin, HOMA‐IR, TAC and MDA.

**FIGURE 4:**
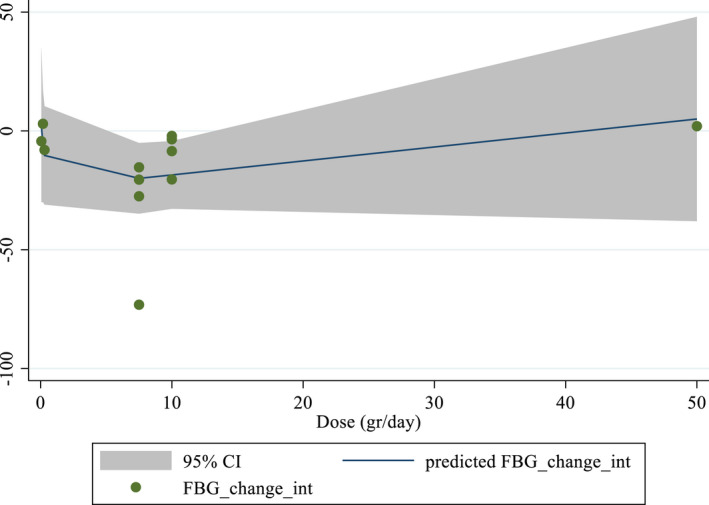
Non‐linear dose–response relations between purslane consumption and absolute mean differences. Dose–response relations between dose (gr/day) and absolute mean differences in FBS (mg/dL).

**FIGURE 5:**
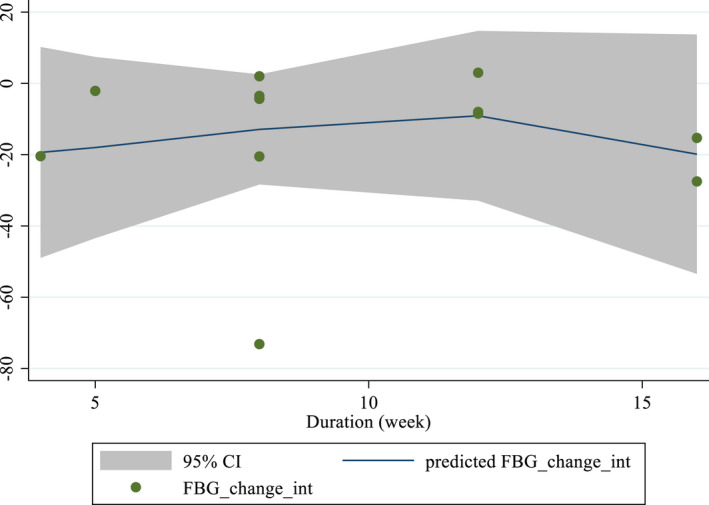
Non‐linear dose–response relations between purslane consumption and absolute mean differences. Dose–response relations between duration of intervention (week) and absolute mean differences in FBS (mg/dL).

**FIGURE 6:**
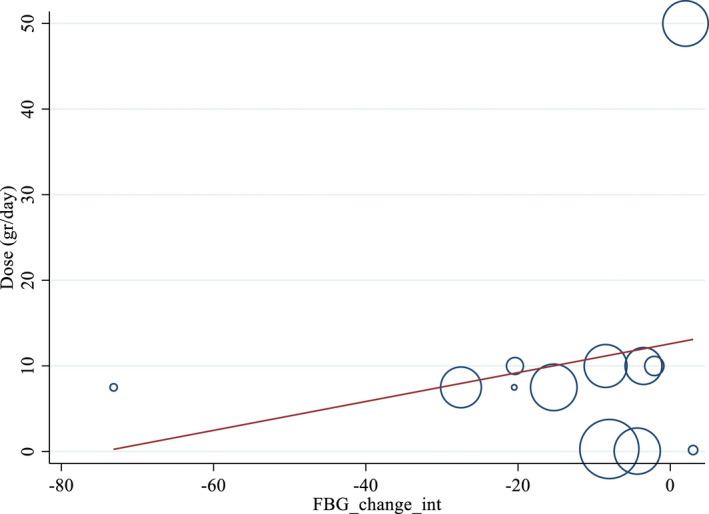
Linear dose–response relations between purslane consumption and absolute mean differences. Dose–response relations between dose (gr/day) and absolute mean differences in FBS (mg/dL).

**FIGURE 7:**
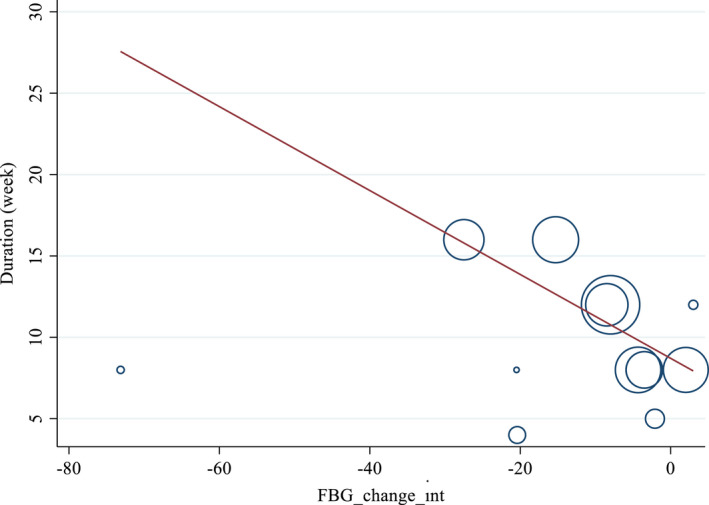
Linear dose–response relations between purslane consumption and absolute mean differences. Dose–response relations between duration of intervention (week) and absolute mean differences in FBS (mg/dL).

#### 3.3.9 Grading of evidence

The GRADE protocol was applied to find the certainty of the evidence (Table 4). The effect evaluation of risk of bias for all factors was downgraded with low quality for severe heterogeneity. The effect assessment of inconsistency for factors like FBS, HbA1c, fasting insulin and MDA was downgraded with low quality for serious and very serious heterogeneity. Results showed that all factors except FBS were downgraded with low quality for serious and very serious heterogeneity. The systematic review and meta‐body analyses of evidence were rated as having moderate overall quality.

**TABLE 4:** GRADE profile: The effects of purslane supplementation on glycemic control and oxidative stress.

Quality assessment						Summary of findings		Quality of evidence
Outcomes	Risk of bias	Inconsistency	Indirectness	Imprecision	Publication bias	Number of intervention/control	WMD (95%CI)	
FBS	Serious limitations^a^	Very serious limitations^b^	No serious limitations	No serious limitations	No serious limitations	904 (448/456)	−8.05 (−12.57, −3.53)	⊕ ⊕ ◯◯ Low
HbA1c	Serious limitations^a^	Serious limitations^c^	No serious limitations	Serious limitations^d^	No serious limitations	167 (85/82)	−0.09 (−0.48, 0.30)	⊕ ⊕ ◯◯ Low
fasting insulin	Serious limitations^a^	Very serious limitations^e^	No serious limitations	Serious limitations^d^	No serious limitations	287 (143/144)	−2.12 (−6.11, 1.87)	⊕◯◯◯ Very low
HOMA‐IR	Serious limitations^a^	No serious limitations	No serious limitations	Serious limitations^d^	No serious limitations	287 (143/144)	−0.14 (−0.45, 0.17)	⊕ ⊕ ◯◯ Low
TAC	Serious limitations^a^	No serious limitations	No serious limitations	Serious limitations^d^	No serious limitations	154 (77/77)	0.24 (0.10, 0.38)	⊕ ⊕ ◯◯ Low
MDA	Serious limitations^a^	Serious limitations^f^	No serious limitations	Serious limitations^d^	No serious limitations	242 (121/121)	−1.23 (−1.59, −0.86)	⊕◯◯◯ Very low

^a^ The most of the studies had low quality.

^b^ The test for heterogeneity is significant for FBS, and the *I* ^2^ is high, 86.1%.

^c^ The test for heterogeneity is significant for HbA1c, and the *I* ^2^ is high, 61.2%.

^d^ The sample sizes less than 400.

^e^ The test for heterogeneity is significant for fasting insulin, and the *I* ^2^ is high, 86.4%.

^f^ The test for heterogeneity is significant for MDA, and the *I* ^2^ is high, 44.8%.

## 4 DISCUSSION

This is the first systematic review and meta‐analysis that investigated the association between purslane consumption, glycemic control, and oxidative stress. This meta‐analysis of 16 trials including 850 participants, showed a relationship between purslane intervention and oxidative stress. We found a significant reduction in FBS, MDA and increase in TAC after purslane consumption. However, no significant effect of purslane was observed on HbA1c, fasting insulin and HOMA‐IR in overall effect size.

Subgroup analysis showed that purslane supplementation of >10 mg/day when the baseline serum FBS is >100 mg/dL has significant beneficial effect on FBS. Also, purslane appeared to be effective on both sexes either with BMI = 25–29.9 or ≥ 30, along the duration of less and more than 8 weeks in controlling FBS concentrations.

According to the current rise in diabetes‐related mortality and morbidity around the world, (Roglic & Unwin, 2010; Shaw et al., 2010), numerous studies have reported the association between diabetes and CVD as the primary cause of death (Fox et al., 2015; Wright et al., 2020). Macrovascular and microvascular conditions accompanied by coronary heart disease, peripheral vascular diseases, stroke, cerebrovascular disease, neuropathy along with lower‐extremity amputations, nephropathy, and retinopathy, are some complications associated with diabetes (Bansal et al., 2006; Gerstein & Werstuck, 2013; Gross et al., 2005; Yun et al., 2008). In addition to above mentioned comorbidities, mental health and cognitive functioning, as well as hepatic, digestive, and musculoskeletal systems are affected by diabetes (Lu et al., 2009). Epidemiologic, clinical, and experimental studies have drawn attention to several therapeutic effects of purslane seeds on pathologic conditions (El‐Sayed, 2011; Sabzghabaee et al., 2014; Shobeiri et al., 2009). Purslane has a broad range of pharmaceutic properties including antidiabetic, neuroprotective, anti‐inflammatory, antioxidant, and anticancer activities (Iranshahy et al., 2017; Rahimi et al., 2019; Zhou et al., 2015). Purslane derives its benefits from polyunsaturated fatty acid and omega‐3 fatty acids, vitamin E, vitamin C, beta‐carotene, alkaloids, flavonoids, polysaccharide, cardiac glycosides, coumarins, and anthraquinone glycosides (PARVIN et al., 2013; Sabzghabaee et al., 2014). There is clear evidence that omega‐3 fatty acids as the major components of purslane have been widely acknowledged for their significant beneficial effects on FBS (García‐López et al., 2016; Khalili et al., 2021; Liu et al., 2022), HbA1C (Khalili et al., 2021), HOMA.IR (Khalili et al., 2021; Liu et al., 2022), and insulin (Liu et al., 2022). Omega‐3 fatty acids have also been linked to a reduction in FBS, insulin, and HOMA‐IR when taken with vitamin E (Asemi et al., 2016; Taghizadeh et al., 2016).

Although the precise mechanism underlying purslane's impact on the glycemic profile is still unclear, some mechanisms have been introduced regarding its beneficial effects including the upregulation of different protein expressions such as, glucose transporter 4 (GLUT‐4), proliferator‐activated receptor (PPAR‐*α*), and PPAR‐*γ* (Jung et al., 2021). Another mechanism which interprets cellular pathway and illustrates that HM‐chromanone, as a component of purslane, is activation of the Phosphoinositide 3‐kinases/ Protein kinase B (PI3K/AKT), Calcium/calmodulin‐dependent protein kinase kinase β‐AMP‐activated protein kinase (CaMKKβ‐AMPK) and Glycogen synthase kinase‐3 (GSK3) α/β pathways. As a result of these activations, glucose uptake and glycogen synthesis occurred in skeletal muscle cells (Park et al., 2021). Jung et al. concluded that purslane supplementation could be used to decrease blood glucose and body fat as well as to prevent and treat diabetes‐related diseases and obesity by reducing weight gain (Jung et al., 2021). A review study revealed that purslane could considerably reduce blood glucose and alleviate lipid profiles in metabolic syndrome patients (Jalali & Rahbardar, 2022). Also, a systematic review of six RCTs, comprising 352 subjects investigating the effect of purslane on blood glucose and lipids showed that purslane can decrease FBS and triglycerides levels. However, it had no significant effect on plasma levels of total cholesterol, LDL, and high‐density lipoprotein (HDL) cholesterol (Hadi et al., 2019). The results of our meta‐analysis supports earlier systematic reviews and meta‐analyses regarding the favorable benefits of purslane on glycemic profile (Hadi et al., 2019; Jalali & Rahbardar, 2022). A study on C57BL/Ksj‐db/db mice indicated that 6‐week supplementation of purslane could significantly reduce the levels of blood glucose, HOMA‐IR and HbA1c (Lee et al., 2020). The lack of sufficient studies on the effects of purslane on glycemic profile may be a contributing factor to the disparity between the findings of the meta‐analyses addressing the effects of purslane on HOMA‐IR and HbA1c.

Continuous elevated glucose levels interfere with many defense mechanisms that inhibit excessive synthesis of reactive oxygen molecules and oxidative stress. A change in the antioxidant defense system would certainly cause a considerable change in the antioxidant enzymes (Seven et al., 2004). MDA is one of the most frequently used biomarker of oxidative stress (Halliwell & Gutteridge, 1986). The extract of purslane along with its antioxidants constituents, such as omega‐3 fatty acids, gallotannins, *α*‐tocopherols, ascorbic acid, apigenin, quercetin, and kaempferol, can mitigate/ameliorate hydrogen peroxide‐induced oxidative DNA damages in human lymphocytes (Behravan et al., 2011). Several investigations supported the view that *α*‐tocopherol as a lipid‐soluble vitamin acts as a lipid peroxyl radical scavenger and prohibits lipid peroxidation chain reactions in the cell membranes (Birben et al., 2012). Moreover, a large number of studies have revealed that purslane has a high omega‐3 fatty acids content, which has been associated with lowering MDA and TAC (Abdollahzad et al., 2009; Badgujar et al., 2015; Boonthongkaew et al., 2021; Grabež et al., 2022). As opposed to our finding, a meta‐analysis and systematic review of 10 intervention trials showed insignificant effect of purslane supplementation on MDA, TAC (Zhu et al., 2021). In vitro studies on purslane's anti‐oxidant mechanisms suggested that the extract could reduce oxidative stress by altering the blood and liver antioxidant enzyme activities, which would result in higher levels of leptin/−actin and liver peroxisome proliferator‐activated receptors (PPAR) a/−actin (Chen et al., 2012).

Purslane has been widely used in traditional medicine (El‐Sayed, 2011). Although none of the studies included in the current investigation reported any significant negative effects related to purslane supplementation, there are still some minor concerns about the high levels of oxalic acid concentrations which may probably lead to malabsorption of some minerals (Bataille & Fournier, 2001). Moreover, oxalic acid can increase the risk of kidney stones (Palaniswamy et al., 2004). Similar to other herbal medicines, pregnant women and children should use purslane with caution (Izzo et al., 2016).

To the best of our knowledge, no previous systematic review and meta‐analysis has specifically evaluated the effect of purslane on glycemic profile and oxidative stress. A notable limitation of our meta‐analysis is the low number of trials that were available for the meta‐analysis which undermines the strength of the conclusion.

In conclusion, this systematic review and meta‐analysis pointed out that purslane significantly lowered the plasma levels of FBS, MDA ameliorated TAC levels. However, it had no meaningful effect on HbA1c, HOMA‐IR, and fasting insulin. Altogether, purslane as powder or capsule can be taken as a supplement to decrease FBS levels, which could minimize the risk of diabetes. However, further well‐designed RCTs are needed to confirm the results of the current study.

## ACKNOWLEDGEMENTS

None.

## CONFLICT OF INTEREST STATEMENT

The authors declared that there is no conflict of interest.

## Data Availability

Data will be made available on request.
